# Pupal Warning Coloration of Three Species of *Cystidia* (Lepidoptera: Geometridae: Ennominae) in Relation to Their Pupation Sites

**DOI:** 10.3390/insects14010038

**Published:** 2022-12-31

**Authors:** Makoto Tsubuki, Fumio Hayashi

**Affiliations:** Department of Biology, Tokyo Metropolitan University, Minamiosawa 1-1, Hachioji, Tokyo 192-0397, Japan

**Keywords:** aposematism, DNA barcoding, lizard predation, predator avoidance, unpalatability

## Abstract

**Simple Summary:**

The evolution of warning (aposematic) colors in insects has been well studied for adult wings and larval bodies. However, there are few reports on the evolution of warning colors in pupae. We hypothesized that pupal coloration of the moth *Cystidia* (Geometridae: Ennominae) is aposematic, and examined the color pattern, pupation site, palatability, and predator’s learning for three species of *Cystidia*. Pupae of *C. couaggaria* and *C. truncangulata* have a yellow surface dotted with black spots, that seems conspicuous for the visually food-searching predators. These two species make the naked pupae on the surface of branches and leaves above the ground, where they are more conspicuous against the predators. Pupae of *C. stratonice* also have a yellow surface with black spots, but the head, thorax, wing pads, and abdominal end are uniformly blackish. This species also differs from the other two species in that it pupates in an above-ground pouch made of leaves by spinning with course silk. In the predation experiment using lizards as one of the potential predators, pupae of all three species were repelled, and repeated biting of pupae increased the lizard’s tendency to avoid them. These results suggest that the conspicuous coloration of unpalatable pupae of *Cystidia* may function as a warning color. However, *C. stratonice* pupating in the leaf pouch may halve the function of the warning color, and the concealing dark color in the pouch may be more developed in this species than in the other two species.

**Abstract:**

Many insects display a cryptic color to avoid detection by predators that search for prey by sight. However, some species with chemicals that predators dislike may display a warning color (aposematism) to predators. The predators can learn easier that the species is unsuitable as prey if the color is more conspicuous. Therefore, it is assumed that the acquisition of the warning color requires not only unpalatability, but also exposure of the color to predators and the ability of predators to recognize and learn it unpalatable. In the moths of the subfamily Ennominae, almost all of genera produce uniformly brown or green pupae, but the pupae of the genus *Cystidia* have conspicuous coloration of yellow background and black spots. In this study, to clarify whether the color of these pupae is the warning color or not, we compared the coloration, pupation site, and palatability among the three species of this genus: *C. couaggaria*, *C. truncangulata*, and *C. stratonice*. Learning by the predators was also examined using lizards as a potential predator of the moths. The results showed that all three species were repelled (unpalatable) by the lizards, and that repeated providing of the pupae to the lizards decreased their willingness to prey on them (probably due to learning). Pupation sites of *C. couaggaria* and *C. truncangulata* were located on the surface of branches and leaves high above the ground, whereas *C. stratonice* pupated in the space of leaves spun with course silk at lower site above the ground. Thus, the conspicuous coloration of pupal *Cystidia* is considered to be a warning color, but the pupae of *C. stratonice* are more blackish than those of the most closely related *C. truncangulata*. The pupal color of *C. stratonice* is likely to have a dual meaning as cryptic and warning colors. The dark colored pupa may be inconspicuous when hidden within the leaf space, but once detected by the predators, the yellow color of the pupa may function as a warning color.

## 1. Introduction

Predation avoidance of the pupal stage in insects has received little attention in comparison to larval and adult stages [1]. In insect life cycles, the pupae are an immobile stage, and the pupation site selection is critical to survive this stage. Pupae of most insect species are cryptic in color and hide under the substrates or in inconspicuous cocoons [1,2,3,4]. However, if insects have toxins or bad odor, they can develop warning coloration (aposematism). Such a prominent color signals to visually hunting predators that the prey is unprofitable through the process of predator’s experience and learning [5]. The pupal color of some insect species is prominent and seems to be warning coloration, for example in *Delias* and *Aporia* of butterflies [6] and *Abraxas grossulariata* (Linnaeus, 1758) of moths [7,8]. However, prominent pupal colors are not always warning colors. Only a few insects have been proven to be warning colors. One is the toxic two-spotted ladybird beetle, *Adalia bipunctata* (Linnaeus, 1758), which has warning colors at all stages of its life cycle [9], and the other is the unpalatable moth, *Ivela auripes* (Butler, 1877), which pupates at the exposed sites such as tree trunk and rock surface above the ground and displays the conspicuous yellow pupal body with seven longitudinal stripes of blackish dots [10].

The moths of *Cystidia* (Lepidoptera: Geometridae: Ennominae) produce colorful pupae exceptionally patterned among Ennominae [11]. We noticed that the colorful pupae of *Cystidia* are exposed on the surface of leaves and twigs above the ground and are likely to be warning coloration. If so, they should be unpalatable for the predators, such as birds, mammals and lizards, that use their vision to search for food. In Japan, three species of *Cystidia* are distributed widely [11]: *C. couaggaria* (Guenée, 1858), *C. truncangulata* Wehrli, 1934, and *C. stratonice* (Stoll, 1782). This genus belongs to the tribe Cystidiini in Ennominae [12,13]. These species are distributed from Far East Russia to Japan [12] and adults appear in early summer [14]. The external morphology of larvae and pupae of these species are described in detail and each species can be identified based on their morphology and color patterns [11,14,15]. Adults fly and mate in the daytime using female sex pheromone (hydrocarbons) [16,17]. Six species of hymenopteran parasitoids are recorded from the pupae of *C. couggaria* [18]. These are the information on *Cystidia* known so far.

In this study, to examine the possibility of warning coloration of these *Cystidia* pupae, first, the pupal size, color, and marking pattern are compared on their phylogenetic tree based on the barcoding COI sequences. Second, pupation sites in the field are compared among the three species. Third, palatability of these pupae is assessed using the lizard as a potential predator. Finally, the observed trend of variation in pupal coloration is discussed in relation to phylogenetic relationships, pupation site selection, and palatability.

## 2. Materials and Methods

### 2.1. Insects

Larvae, pupae and adults of *C. couaggaria* were collected from a forest at Hino, western Tokyo, in May and June 2020. They were placed individually in a plastic cup (130 mm in diameter, 55 mm in depth) and kept at natural temperatures and daylength. Leaves of the tree *Armeniaca mume* (Rosaceae) were given to the larvae. Larvae of *C. truncangulata* were collected from a forest at Moriya, Ibaraki Prefecture, on 24 April 2021, and reared at natural temperatures and daylength, giving leaves of the tree *Celastrus orbiculatus* (Celastraceae). Field-caught pupae from the same locality on 24 May 2020 and adults from Ueda, Nagano Prefecture, on 24 June 2020, were also used for experiments. Larvae, pupae and adults of *C. stratonice* were collected from a forest at Ueda, Nagano Prefecture, in June 2020. The larvae were reared at natural temperatures and daylength, giving leaves of *C. orbiculatus*.

### 2.2. Morphological Measurements

**Figure 1:**
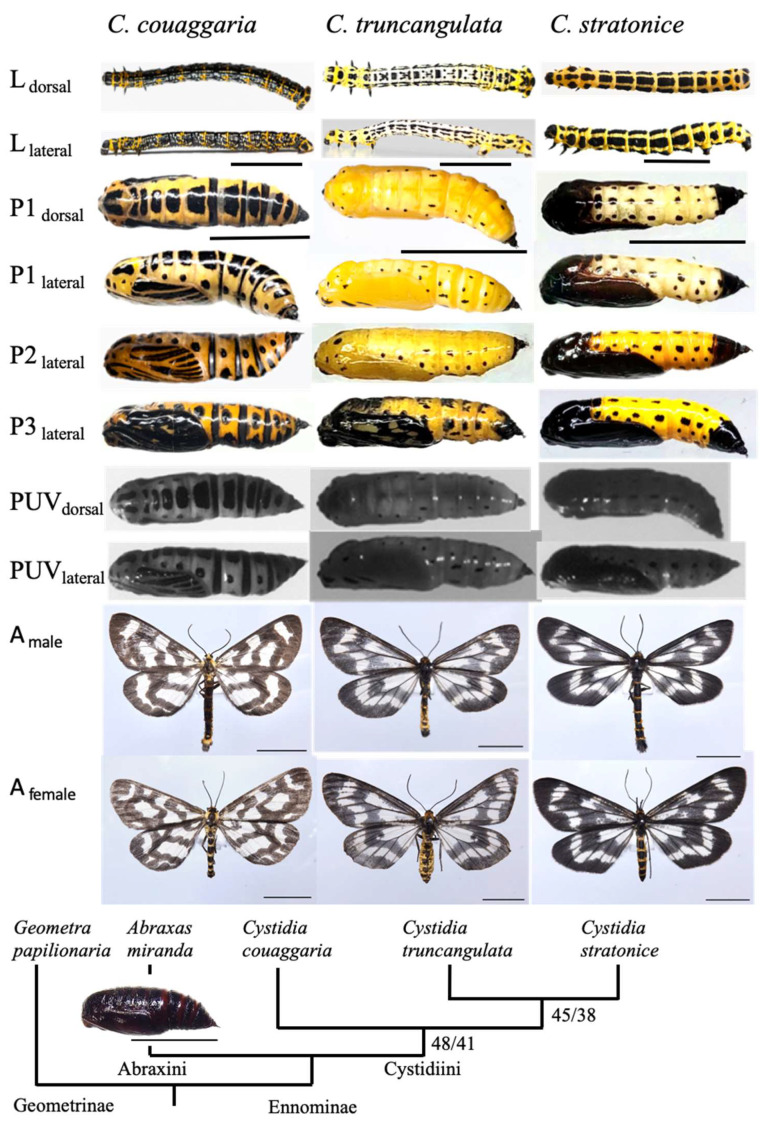
Larvae (L), pupae (P), and adults (A) of the three species of *Cystidia* moths; *C. couaggaria*, *C. truncangulata*, and *C. stratonice*. Each photograph shows the dorsal or lateral view under visible and ultraviolet (UV) light. In pupal stage, P1–P3 shows the pupal color patterns in the day of pupation, 6th or 7th day since pupation, and 1 day before adult emergence. The DNA barcoding result is drawn for three species of *Cystidia* and the outgroup *Abraxas miranda* (JN087399) belonging to the tribe Abraxini of the subfamily Ennominae and *Geometra papilionaria* (GU655815) belonging to the subfamily Geometrinae. The numerals near the branches present % probabilities in the neighbor-joining/maximum likelihood methods. Scale bars show 10 mm.

Body size were measured using the free software, ImageJ (version 1.53e; National Institutes of Health, Bethesda, MD, USA), based on the photographs of randomly chosen 6 final-instar larvae, 11 pupae, 5 male adults, and 5 female adults of *C. couaggaria*; 6 final-instar larvae, 25 pupae, 8 male adults, and 10 female adults of *C. truncangulata*; 8 final-instar larvae, 10 pupae, 6 male adults, and 4 female adults of *C. stratonice*. We measured the body length (from the front of head to the body end) and width (at the widest part of body) of larvae and pupae from the dorsal side, and the forewing length (from base to the tip) of adults (Figure 1).

Visual pigments of the potential predators such as birds and lizards can absorb ultraviolet light [19,20]. Therefore, to know the pupal color patterns under ultraviolet light, the pupal photographs of the three species of *Cystidia* were taken using an ultraviolet filter (U330; HOYA Co. Ltd., Tokyo, Japan).

### 2.3. DNA Barcoding

Legs of the dried adult specimens of *C. couaggaria* collected at Hino, western Tokyo, and *C. truncangulata* and *C. stratonice* both collected at Ueda, Nagano Prefecture, were used for DNA barcoding and molecular phylogenetic analysis. Total genomic DNA was extracted from these 3 individuals, using a DNeasy Blood and Tissue Kit (Qiagen, Hilden, Germany). The mitochondrial cytochrome c oxidase subunit I (COI) gene fragment was amplified using Ex Taq^®^ (TaKaRa, Tokyo, Japan) by the primer set forward 5′-TTATTTTTGGAATTTGAGC-3′ and reverse 5′-CCTGTTAATCCTCCTACTGT-3′ [21]. The PCR reaction mix (total volume 10 µL) contained 1.0 µL 10× Ex Taq Buffer, 0.8 µL 25 mM dNTP mix, 0.5 µL of each the forward and reverse primer (10 pM), 0.05 µL Taq polymerase, 6.15 µL distilled deionized water, and 1.0 µL template DNA. The PCR protocols implemented using a T100™ thermal cycler (Bio-Rad, Hercules, CA, USA) were as follows: an initial 3 min denaturing step at 94 °C; 35 cycles of 20 sec at 94 °C, 20 sec at 50 °C, and 30 sec at 72 °C; with a final 5 min extension at 72 °C. The PCR products were purified with illustra™ ExoProStar™ 1-Step (GE Healthcare, Buckinghamshire, UK) and sequenced using BigDye^®^ Terminator ver. 3.1 (Applied Biosystems, Foster City, CA, USA) on an ABI 3130xl Genetic Analyzer (Applied Biosystems).

Direct sequencing data were aligned with the outgroup data using MEGA X [22]. The sequences were aligned to 585 bp without gaps (GenBank accession numbers LC742382 in *C. couaggaria*, LC742380 in *C. truncangulata*, and LC742381 in *C. stratonice*) and the outgroup *Abraxas miranda* Butler, 1878 (JN087399) [23] belonging to the tribe Abraxini of the subfamily Ennominae and *Geometra papilionaria* (Linnaeus, 1758) (GU655815) [24] belonging to the subfamily Geometrinae. Phylogenetic trees were constructed by the neighbor-joining (NJ) method based on p-distance (bootstrap replication = 1000) and maximum likelihood (ML) estimation (bootstrap replication = 1000) based on the General Time Reversible model following the gamma distribution with invariant sites using MEGA X [22]. The best-fit nucleotide substitution models were estimated based on the corrected Akaike’s information criterion (AICc) [25] using MEGA X [22].

### 2.4. Pupation Site Selection

**Figure 2:**
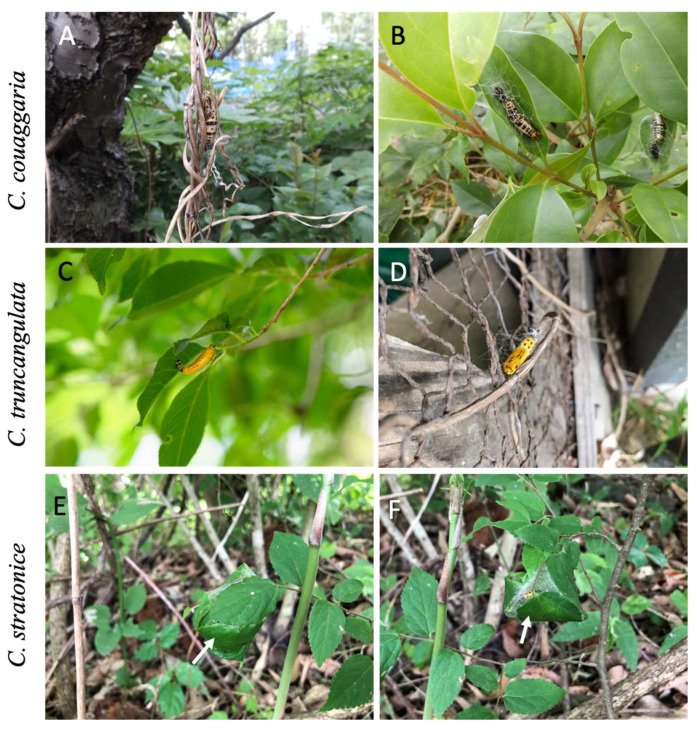
Pupation sites in the field of the three species of *Cystidia* moths; *C. couaggaria* (**A**,**B**), *C. truncangulata* (**C**,**D**), and *C. stratonice* (**E**,**F**). They pupate on the surface of twigs (**A**,**C**), upside a leaf (**B**), underside a leaf (**C**), and within the leaves stitched with silk, showing by white arrows (**E**,**F**).

We searched for prepupae, pupae and pupal exuviae of *C. truncangulata* and *C. stratonice* in a forest at Ueda, Nagano Prefecture, on 31 May and 2 June 2020 and 13 June 2021. Those of *C. couaggaria* were collected from a forest at Ichikawa, Chiba Prefecture, on 13 May 2021. We recorded the location by height from the ground beginning at 0 m if located on the soil surface. Each pupation site was recorded by distinguishing the situation into five categories; on twig surface, on artificial substrate surface (wire net and concrete pillar), upper surface of a leaf, underside of a leaf, and within leaves stitched together with silk (Figure 2).

### 2.5. Assessment of Palatability

A total of 36 (#1–36) lizards, *Plestiodon finitimus* Okamoto et Hikida, 2012, were used as a potential predator. These lizards were caught at Inagi, Tama, Hino, and Hachioji Cities, western Tokyo, in May 3, 7, 13, 27, 29, and June 15, 24, 26, 2020 and April 12, 16, 18, 2021. The mean snout to vent length (SVL) of them was 60.4 mm (SD = 4.1, *N* = 36) and total tail length was 76.3 mm (SD = 22.0, *N* = 36). They were kept individually in plastic cages (200 mm × 350 mm, 200 mm in depth) at 17–33 °C with natural daylength. Each cage was filled up to 10 mm with a mixture of peat moss and red ball soil in a 1:1 ratio and an unglazed shelter. Water was supplied constantly in a cup (90 mm in diameter, 50 mm in depth). Two mealworms (larvae of *Tenebrio molitor* Linnaeus, 1758) were fed during the 3-h basking period with artificial light every other day.

Feeding experiments were conducted for lizards maintained in the cage for at least one week. To test palatability of larval, pupal, and adult *C. couaggaria*, after 30-min basking, one larva was given to #1–3 lizards daily for five consecutive days (1st- to 5th-day trials). Similarly, one pupa was given to #4–6 lizards, one male adult to #7–9 lizards, and one female adult to #10–12 lizards daily for five days. This 5-day feeding experiment was conducted also in *C. truncangulata* using #13–24 lizards and in *C. stratonice* using #25–36 lizards. The feeding willingness of each lizard was checked by giving one mealworm just before the 1st-day trial and by giving three mealworms just after the 5th-day trial. To prevent the adult moth from flying out of the cage bottom, adults were given after cutting the forewings partly at their edges. Pupae were suspended by a thin black polyester thread (0.15 mm in diameter) and presented to the lizards. All tests were terminated if there was no approach to the prey for 20 s. Lizard behavior was recorded with a video camera (12 MPx Camera, Apple Inc., Cupertino, CA, USA) of the Apple iPhone 8. The lizard’s behavior was discriminated into seven categorized sequences: no response (0), heading toward prey (1), licking prey (2), pecking prey with their mouthparts (3), biting prey but immediately releasing it (4), swallowing prey but regurgitating it (5), and complete feeding (6). Once the lizards were used for the 5-day feeding experiment, they were released to the capture sites in the field.

### 2.6. Statistics

Values are shown as mean ± standard deviation (SD) with sample size (*N*). To compare the height of pupation sites from the ground surface among the three species, the Kruskal–Wallis test and the following Steel-Dwass multiple comparison test were used. Two-way analysis of variance (two-way ANOVA) was used to test differences in the lizard body size (SVL) among the feeding experiment groups which differ in the developmental stages and species of *Cystidia*.

## 3. Results

### 3.1. Morphological Comparison

Larvae are elongated in the three species, but the color patterns differed among the species (L in Figure 1). Body length of the last-instar larvae is 32.1 ± 4.5 mm (*N* = 6) in *C. couaggaria*, 30.6 ± 5.7 mm (*N* = 6) in *C. truncangulata*, and 31.8 ± 3.3 mm (*N* = 8) in *C. stratonice*. Their body width is 3.3 ± 0.7 mm (*N* = 6), 3.2 ± 0.4 mm (*N* = 6), and 3.5 ± 0.4 mm (*N* = 8), respectively.

Pupae of the three species similarly have blackish dotted lines longitudinally on the yellow body (P in Figure 1). However, the blackish markings differ among the species; larger marking in *C. couaggaria*, smaller dotted marking in *C. truncangulata*, and totally blackish in the head, thorax including wing pads, and caudal segments of the abdomen in *C. stratonice*. Such blackish markings are kept during the pupal stage, except for the stage near adult emergence at which the adult wing marking is formed in wing pads (P1–P3 in Figure 1). The blackish marking patterns do not differ between the photographs taken under visible and ultraviolet light conditions (PUV in Figure 1). The pupal length is 21.0 ± 1.3 mm (*N* = 11) in *C. couaggaria*, 17.7 ± 3.0 mm (*N* = 25) in *C. truncangulata*, and 18.2 ± 1.8 mm (*N* = 10) in *C. stratonice*. Their width is 5.3 ± 0.4 mm (*N* = 11), 4.8 ± 1.1 mm (*N* = 25), and 4.6 ± 0.4 mm (*N* = 10), respectively.

The adult commonly has white and black stripes on the wings and yellow and black bands on the abdomen. Male forewing length is 24.0 ± 1.0 mm (*N* = 5) in *C. couaggaria*, 25.4 ± 0.9 mm (*N* = 8) in *C. truncangulata*, and 26.7 ± 0.8 mm (*N* = 6) in *C. stratonice*. Female forewing length is 22.2 ± 1.5 mm (*N* = 5), 26.8 ± 1.8 mm (*N* = 10), and 25.9 ± 0.7 mm (*N* = 4), respectively.

Phylogenetic relationships of DNA barcoding results suggest that *C. truncangulata* and *C. stratonice* are more closely related among the three species of *Cystidia* (the tribe Cystidiini), although not supported strongly (Figure 1). These relationships are also reflected in morphological differences. The dotted black marking pattern on the yellow pupa and the blackish striped pattern on wings are closely similar between *C. truncangulata* and *C. stratonice*, but such patterns differ in *C. couaggaria*. In the former two species, however, the areas of pupal blackish marking are much wider in *C. stratonice*. The pupae of *Abraxas miranda* belonging to the different tribe Abraxini is entirely brown (Figure 1). This pupa was obtained by rearing a field-caught last-instar larva in Hachioji, western Tokyo, on 9 October 2021, and pupated on the bottom of the rearing cage.

### 3.2. Pupation Site

**Figure 3:**
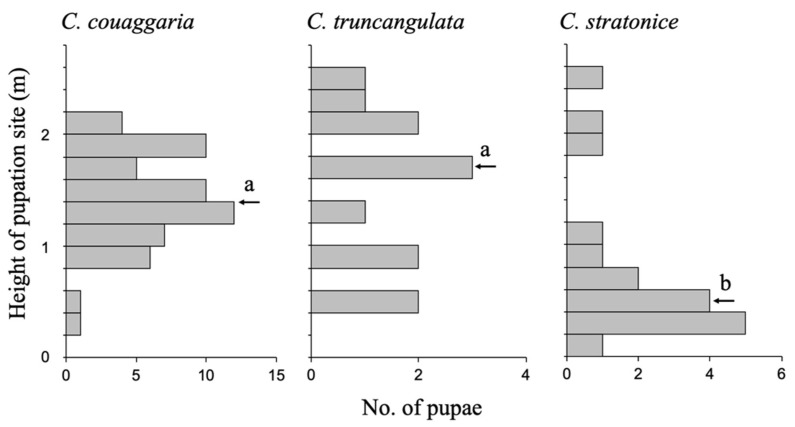
Height of the pupation sites from the ground surface of the three species of *Cystidia* moths; *C. couaggaria* (*N* = 56), *C. truncangulata* (*N* = 12), and *C. stratonice* (*N* = 17). Arrows indicate medians and the same alphabet denotes insignificant difference (*p* > 0.05) in the Steel-Dwass multiple comparison test after the Kruskal–Wallis test (*p* < 0.001).

All pupae were found above the ground in the field (Figure 2). The median of pupation height was 1.4 m in *C. couaggaria*, 1.7 m in *C. truncangulata*, and 0.5 m in *C. stratonice* (Figure 3). The former two species pupated higher sites than the latter species (Kruskal–Wallis test, *χ*^2^ = 14.7, df = 2, *p* < 0.001). Pupae of *C. couaggaria* and *C. truncangulata* were exposed on the surface of twigs, leaves, and artificial wire mesh fences and stone blocks, whereas those of *C. stratonice* were hidden within the leaves roughly stitched together with silk (Table 1, Figure 2).

### 3.3. Palatability

The lizard body size in SVL did not differ among the feeding experiment groups (Table 2: two-way ANOVA; developmental stage, *F*_3, 35_ = 0.27, *p* = 0.85; species, *F*_2, 35_ = 1.65, *p* = 0.21; interaction, *F*_6, 35_ = 1.04, *p* = 0.43). All mealworms given at the 1st and 5th days were eaten by the lizards, whereas all stages of *C. couaggaria*, *C. truncangulata*, and *C. stratonice* were not eaten, suggesting that these moths are unpalatable for the lizards during their development (Table 2). In the 1st- to 3rd-day of feeding trials, most lizards showed the feeding sequences of pecking with their mouthparts (3) and biting (4), but after that such positive responses to the prey did little occur (Table 2). Thus, it is likely that lizards learn prey unpalatability if the moth of the same developmental stage were repeatedly given.

## 4. Discussion

Bodies and wings of most insect species resemble fresh or dead leaves and tree trunks and branches, which is a phenomenon known as cryptic coloration to avoid detection by the predators that use their vision to search for food [5]. Almost all pupae of 119 genera of Japanese Ennominae are known to be uniformly brown (pale, reddish, or dark brown) or green in *Auaxa sulphurea* [11]. The brown and green pupae may be cryptic to the background color. Among these 119 ennomine genera, pupae of the three species of *Cystidia* are reported to be exceptionally yellow with some black spots [11]. We confirmed this fact that pupae of *C. couaggaria*, *C. truncangulata*, and *C. stratonice*, are similar to each other in shape, size, color, and marking pattern (Figure 1). The marking pattern did not differ between visible and ultraviolet lights (Figure 1), suggesting that there was a similar image for birds and lizards that can use ultraviolet vision. The phylogenetic analysis suggests that *Cystidia* is monophyletic and *C. truncangulata* and *C. stratonice* are more closely related than *C. couaggaria* (Figure 1). However, there are some species-specific differences in pupal marking patterns (Figure 1). The black markings of *C. couaggaria* are usually larger and form at least three bands in the abdomen. The pupae of *C. truncangulata* have a smaller black spot and are widely yellowish, whereas the pupae of *C. stratonice* are more blackish throughout the pupal stage.

The conspicuous color common to *Cystidia* species is likely to be warning coloration. If the insect is equipped with defenses against predators such as toxins or inappetence, selection may favor warning colorations [5]. Warning coloration is a widespread strategy to alert predators about such prey unprofitability. The success of this strategy partly depends on predators being able to learn and recognize certain signals as indicators of toxicity by encountering frequently [26]. Thus, to discuss the possibility of pupal warning coloration, we need information of encountering rates to the predators, unpalatability of the pupae, and the ability of learning by the predators.

All three species of *Cystidia* pupate above the ground (Figure 2). Pupae of *C. couaggaria* and *C. truncangulata* are exposed on the surface of twigs, leaves, and artificial materials such as wire mesh fences and stone blocks, but those of *C. stratonice* are all hidden inside the leaves roughly spun with silk (Table 1). The height of pupation sites from the ground surface is greater in the former two species than the latter (Figure 3). Thus, the pupae of *C. couaggaria* and *C. truncangulata* seem to be more exposed to the predators and detected by the predators more frequently than those of *C. stratonice*. Most insects that pupate above the ground are cryptic in color that serve as camouflage to deceive the eyes of predators [2,27,28]. The exceptional examples are the toxic two-spotted ladybird beetle *Adalia bipunctata*, which has warning colors at all developmental stages [9], and the unpalatable moth *Ivela auripes* [10]. Warning colors evolved to warn predators that the prey is not profitable [29,30]. The benefit of conspicuous coloration strongly promotes predator learning in association with repellency [29,30]. The pupae of *C. couaggaria* and *C. truncangulata* are exposed to predators above the ground and their color is conspicuous just as the pupae of the ladybird beetle *A. bipunctata* and the moth *I. auripes*. In contrast, the pupae of *C. stratonice* are inside of the roughly spun leaves at the lower site above the ground. The pupal color of *C. stratonice* is likely to have a dual meaning as cryptic and warning colors. The more blackish pupa may be inconspicuous when hidden within the leaf space, but once detected by the predators, the yellow and black color pattern of the pupa may function as a warning color. To understand such interspecific differences in pupal colors, further attention should be paid to the effects of the pupation site environment on the predation intensity.

All developmental stages of larvae, pupae and adults of the three species of *Cystidia* are unpalatable for the lizards (Table 2). Under the experimental conditions, the lizards became to ignore the pupae with their feeding experience. Such a tendency suggests that they learn unpalatability of the pupae by their conspicuous coloration. Thus, the unpalatable pupae potentially show warning colors. The chemical analysis has never been conducted for *Cystidia*, but other ennomine moths, such as *Arichanna gaschkevitchii* (=*A. jaguararia*) and *Abraxas grossulariata*, are reported strongly toxic [7,31,32]. Adult *A. gaschkevitchii* includes grayanoid diterpenes that are acquired by larval host plant *Pieris japonica* (Ericaceae) and are effective in escaping from predation by birds and geckos. The adult of this moth exhibits an aposematic color pattern, suggesting the function of a warning color [32]. However, this moth pupates in the soil on the ground surface [15] and the pupae are uniformly brown [11]. Conspicuously colored adults and larvae of *A. grossulariata* include a bitter tasting cyanoglucoside, sarmentosin, that is effective in escaping from the predators such as birds, lizards, and frogs [7]. The pupae of this moth also exhibit an aposematic color pattern of a wasp-like yellow and black stripes and are visible from the loose cocoon above the ground, suggesting the function of a warning color [7,8]. The adults of *Cystidia* of the present study fly in the daytime, and the pupae are also exposed to the predators on the branches and leaves above the ground, excluding *C. stratonice* with a partially conspicuous color. Thus, the development of warning coloration of lepidopteran pupae seems to be affected by pupation site selection, which is exposed to the predators or not, after acquisition of unpalatability. This is a testable hypothesis of the warning color evolution of the pupal stage in insects.

## 5. Conclusions

Pupae of the geometrid moth *Cystidia* were unpalatable to lizards, one of the potential predators. When the pupae were repeatedly given to the lizards, they gradually ignore the pupae as prey. This suggests that the lizards learn unpalatability of the pupae through experience. Pupae were yellow with black spots, making them conspicuous. Furthermore, pupae are located on the surface of tree branches and leaves above the ground, where they are exposed to predators that search for prey with their eyes. These results indicate that the conspicuous color of *Cystidia* pupae functions as a warning color. However, the pupae inside the leaves spun with silk show more blackish marking pattern on the yellow body than the exposed pupae. This tendency to darken is thought to have two effects: one was to conceal the pupae to make it inconspicuous within the leaves, and the other was to serve as a warning color once predators detect them. To understand the interspecific differences in pupal colors, further attention should be paid to the relationship between the environment of the pupation site and pupal coloration.

## Figures and Tables

**Table 1:** Pupation sites of the three species of *Cystidia* moths; *C. couaggaria*, *C. truncangulata*, and *C. stratonice* in the field. The number of pupae found at five categories of pupation sites is shown (see Figure 2).

Pupation Site	On Twig Surface	On Artificial Substrate Surface	Upside a Leaf	Underside a Leaf	In Leaves Stitched Together with Silk	Total
*C. couaggaria*	8	1	47	0	0	56
*C. truncangulata*	4	5	0	3	0	12
*C. stratonice*	0	0	0	0	17	17

**Table 2:** The lizard feeding behaviors in the five-day feeding trials of individual lizards (#1–36), when given larvae (L), pupae (P), adult males (Am), and adult females (Af) of the three species of *Cystidia* moths: *C. couaggaria*, *C. truncangulata*, and *C. stratonice*. Feeding behavior is shown by the six sequences to which lizards reach: no response (0), heading toward prey (1), licking prey (2), pecking prey with their mouthparts (3), biting prey but immediately releasing it (4), swallowing prey but regurgitating it (5), and complete feeding (6). Categories (5) and (6) are not observed, and more active responses (3) and (4) are shaded. SVL shows the lizard snout to vent length in mm.

Lizaed ID	#1	#2	#3	#4	#5	#6	#7	#8	#9	#10	#11	#12
Lizaed SVL (mm)	59	55	64	55	64	69	60	66	60	55	61	59
Stage of *C. couaggaria*	L	L	L	P	P	P	Am	Am	Am	Af	Af	Af
1st day	4	4	2	3	4	4	4	4	4	4	4	4
2nd day	4	0	0	4	4	2	4	4	2	3	2	2
3rd day	4	1	1	2	0	2	4	4	4	1	2	0
4th day	3	2	1	1	0	0	0	0	0	0	1	0
5th day	2	2	0	0	0	0	0	0	1	0	2	2
**Lizaed ID**	**#13**	**#14**	**#15**	**#16**	**#17**	**#18**	**#19**	**#20**	**#21**	**#22**	**#23**	**#24**
**Lizaed SVL (mm)**	**64**	**59**	**57**	**58**	**58**	**54**	**57**	**55**	**58**	**64**	**61**	**60**
**Stage of *C. truncangulata***	**L**	**L**	**L**	**P**	**P**	**P**	**Am**	**Am**	**Am**	**Af**	**Af**	**Af**
1st day	4	4	4	4	4	4	4	4	4	4	4	4
2nd day	2	2	2	1	2	4	4	4	4	4	4	4
3rd day	2	2	1	0	0	1	1	4	4	0	4	3
4th day	0	0	1	1	0	1	1	2	4	0	0	0
5th day	0	1	2	2	0	2	1	1	2	0	0	0
**Lizaed ID**	**#25**	**#26**	**#27**	**#28**	**#29**	**#30**	**#31**	**#32**	**#33**	**#34**	**#35**	**#36**
**Lizaed SVL (mm)**	**66**	**65**	**61**	**61**	**58**	**60**	**70**	**58**	**55**	**64**	**68**	**57**
**Stage of *C. stratonice***	**L**	**L**	**L**	**P**	**P**	**P**	**Am**	**Am**	**Am**	**Af**	**Af**	**Af**
1st day	4	4	4	4	4	4	4	3	2	3	1	4
2nd day	2	4	2	2	2	2	4	4	2	1	1	4
3rd day	2	4	2	0	1	2	4	2	0	0	0	2
4th day	2	1	0	0	1	1	2	2	1	0	0	1
5th day	2	4	1	1	1	1	1	3	0	1	1	1

## Data Availability

The data presented in this study are available on request from the authors.
